# Efficient maltotriose fermentation through hydrolysis mediated by the intracellular invertase of *Saccharomyces cerevisiae*

**DOI:** 10.1186/1753-6561-8-S4-P181

**Published:** 2014-10-01

**Authors:** Victor Ribeiro de Godoy, Gabriela Muller, Bóris Stambuk

**Affiliations:** 1 Universidade Federal de Santa Catarina, Florianópolis, SC, Brazil

## Background

It is well known that in the yeast *S. cerevisiae *the sugars sucrose and maltose/maltotriose are metabolized by different pathways: sucrose is hydrolyzed by extracellular invertase (encoded by *SUC *genes), while maltose and maltotriose are actively transported into the cell and hydrolyzed by intracellular α-glucosidases (both proteins encoded by the *MAL *genes). Nevertheless, several reports have shown that some *SUC *genes can be located proximal to *MAL *genes at the telomeres of different chromosomes. Furthermore, the *SUC *genes also allow the synthesis of an intracellular form of invertase, an enzyme with no obvious function in yeasts [1]. We have already shown that sucrose can be metabolized by yeast cells through *MAL*-encoded transporters and α-glucosidases.

## Methods, results and conclusions

Now, our results will show that maltotriose can be efficiently fermented by *S. cerevisiae *cells through its active transport mediated by the *AGT1 *permease, a *MAL *transporter required for maltotriose utilization [2,3], and its intracellular hydrolysis mediated by the cytoplasmic invertase. The Brazilian industrial fuel-ethanol strain CAT-1 cannot ferment maltotriose efficiently due to a defective promoter of the *AGT1 *gene [4]. To increase maltotriose fermentation by this strain, we placed a strong promoter (P_GPD_) in the *AGT1 *gene of strain CAT-1, generating strain GMY05. While the *AGT1 *gene was indeed over-expressed in this strain (measured by real-time PCR and a specific transport assay), maltotriose was still not fermented efficiently. However, when we over-expressed the intracellular form of invertase, by replacing the signal sequence of the *SUC2 *gene with the strong P_PGK _promoter, the resulting *iSUC2 *strain GMY08 fermented maltotriose efficiently. Using conditions were the *MAL*-encoded α-glucosidases would not be expressed, we could show that the intracellular form of invertase hydrolyzes maltotriose efficiently (but not maltose or *p*-nitrophenyl-α-glucoside), specially at the cytoplasmic pH of 7.0. Under the same conditions we purified the intracellular invertase by ion-exchange chromatography, and the identity of the enzyme confirmed by mass spectrophotometry. With the purified enzyme we performed enzymatic tests that corroborated our previous analysis, showing that intracellular invertase hydrolyzes maltotriose. Thus, our results indicate an unexpected overlap in sucrose-maltotriose metabolism by yeast cells, showing that the intracellular invertase allows efficient maltotriose hydrolysis, and offers new approaches that can be applied to optimize several industrial fermentation processes that use starch hydrolysates, including production of distilled beverages, brewing and backing.
